# 

**Published:** 1914-06-13

**Authors:** 


### Medical Appointments.

Mr. John F. Herbert, B.Sc., M.B., B.Ch.I., has been appointed assistant house surgeon at North Ormesby Hospital, Middlesbrough.

Mr. T. P. McMurray, M.B., B.Ch. Q.U. Bel., late assistant surgeon to the Liverpool Hospital for Children, has been elected honorary assistant surgeon to the David Lewis Northern Hospital, Liverpool. He formerly held the post of house surgeon at the Royal Southern Hospital. Mr. J. H. Rawlinson, M.B., Ch.B. Liv., has also been appointed honorary assistant surgeon at the David Lewis institution. He was formerly house surgeon, and is now surgical tutor at the David Lewis Northern Hospital.

---

Mr. R. Brooks, secretary of the committee of the North Mid-Tyne Governors in connection with a local undertaking, has been appointed a life governor of the Newcastle Royal Victoria Infirmary.

Mr. David Morrison, a representative of the Workmen Governors of the South Tyne district, has had his nomination to serve on the house committee of Newcastle Royal Victoria Infirmary confirmed.

Lord Knutsford, who had a serious operation in the middle of February, has been away from the hospital until last week, when he was welcomed at the quarterly court on Wednesday, and the committee acknowledged their indebtedness to Mr. Douro Hoare, who throughout this period has acted as chairman.

### Gifts and Bequests.

Mrs. Frances Mary Rayson, of Leamington, has left £1,000 to Leicester Infirmary.

Miss Jane Whipp, of Prestwich, Lancs., has left £300 to the Bury Dispensary Hospital.

The Treasurers of the Middlesex Hospital have received an anonymous donation of £100 through Sir Alfred Pearce Gould.

Alderman Charles Nathaniel Brown, of Great Yarmouth, has left £400 to the Great Yarmouth General Hospital.

The King has sent £10 as an annual subscription to the funds of the Central London Throat and Ear Hospital, Gray's Inn Road.

The sale of work which was recently held by the Ladies' Committee of the Chelsea Hospital for Women for the rebuilding fund realised £350.

Miss Katharine Alice Crockett, of San Remo, Italy, some time of Devizes, Wilts, has left £500 to St. Joseph's Home for the Dying, Hackney, and £500 to the Distressed Gentlefolks Aid Association, London.

The Chelsea Hospital for Women has received from the Trustees of Smith's (Kensington Estate) Charity £500 towards its rebuilding fund and £100 towards the funds of its convalescent home at St. Leonards-on-Sea.

Miss Sarah Anne Adams, of Coventry, who left estate of the gross value of £509,889, has left £1,000 each to the Walsall Cottage Hospital, the Birmingham General Hospital, and the Coventry and Warwickshire Hospital.

Mrs. Edna Howarth, of Windermere, has left £750 and her five-diamond hoop ring to her companion, Bertha Jones; all other her jewellery and personal effects and, after other bequests, the residue of her property upon trust for her granddaughter, Lily Edna Lorriman Lorriman, and her issue, whom failing the ultimate residue to provide a wing in the Manchester Children's Hospital, Pendlebury, to bear the name "Edna Howarth" in perpetuity.

### BEQUESTS TO THE COVENTRY HOSPITAL.

The late Miss Matilda Gardner has bequeathed to the Coventry and Warwickshire Hospital the sum of £1,000. The hospital has also received £100 from the executors of the late Mr. F. S. O'Brien, which, in accordance with the terms of the will, is paid in the name of the late Mr. O'Brien's son, Mr. Edgar O'Brien.

### PROFESSOR KILLIAN AT BRISTOL.

On Monday in last week, June 1, Professor Killian, the eminent Berlin surgeon, paid a visit to the Bristol Royal Infirmary to meet the honorary medical and surgical staffs of that institution and of the General Hospital to demonstrate before them his methods of diagnosis and treatment of throat cases. A visit was paid to the operating theatre at the old infirmary, where several operations were performed, at which Professor Killian explained his methods.

RATES OF SUBSCRIPTION: Twelve months, 6/6; Six months, 4/-; Three months, 2/-, post free to any address in the United Kingdom. Abroad, Twelve months, 8/8; Six months, 5/-; Three months, 2/6, post free.—Address The Subscription Dept., "THE HOSPITAL," The Hospital Building, 28/29 Southampton Street Strand, London, W.C.

